# Design of a Low-Cost Ultra-Wide-Band Radar Platform

**DOI:** 10.3390/s20102867

**Published:** 2020-05-18

**Authors:** Marko Malajner, Danijel Šipoš, Dušan Gleich

**Affiliations:** Faculty of Electrical Engineering and Computer Science, University of Maribor, Koroška Cesta 46, 2000 Maribor, Slovenia; danijel.sipos@um.si (D.Š.); dusan.gleich@um.si (D.G.)

**Keywords:** ultra-wide-band, pulse radar, ground penetrating radar, sampling mixer, pulse generator

## Abstract

This paper proposes an improved design of a pulse-based radar. An improved design of a pulse generator is presented using step recovery diodes and a signal mixer for the received signal. Two-step recovery diodes produce pulses of 120 ps in duration. A pulse generator is improved by removing the negative power supply, resulting in a reduced number of electronic pulses. A sampling mixer at the receiver’s site receives the generated signal and stretches it from picoseconds into microseconds. The improved pulse generator is also used in the sampling mixer as a strobe pulse generator, which makes the sampling mixer much simpler. The stretched signal is then sampled by a low sample rate using an analog to digital converter. The proposed radar design achieves up to 8 GHz bandwidth and an equivalent receiving sample rate of about 100 GSa/s. The radar is controlled using a software-defined radio called Red Pitaya, which is also used for data acquisition. The proposed radar design uses widely available commercial components, which makes radar design widely available with low cost implementation.

## 1. Introduction

Ultra-wide-band (UWB) radars produce very short radio-frequency (RF) pulses in the range of a sub-nanosecond order and are used for sensing and imaging applications. UWB pulses have good spatial resolution and enable penetration in dielectric materials. A UWB radar, on the receiver side, measures the reflected signals that arise due to the difference in the electrical properties between the observed object and the surrounding environment [1]. Radars in the UWB domain have a wide range of uses, from military applications, like buried land mine detection, to commercial applications, like medical imaging, ground penetration radars, and material characterization [2,3].

The UWB radar systems differ in operation principles and system performances. Basically, UWB radar can be divided into two groups: (i) pulse based radars (time domain) and (ii) radars based on frequency modulations (frequency domain), such as the stepped frequency continuous wave (SFCW) [4,5] or the frequency modulated continuous wave (FMCW) [6]. The FMCW radar method requires the generation of a continuous linear sweep, which is generated mostly using a voltage controlled oscillator (VCO). The SFCW method generates frequency sweeping in steps, which means that multiple VCOs can be used with a combination of frequency dividers to set the desired frequency for each step. On the receiver side, these radars use super-heterodyne architecture [7]. The frequency domain radio sweeps each frequency separately, and this take some time, meanwhile, the pulse radar transmits a wideband of frequencies in one shot. However, frequency domain radars have a much more complicated architecture and signal processing in comparison to pulse radars.

The principal UWB pulse radar consists of a transmitter with a pulse generator, a receiver with a pulse detector, antennas, and a signal processing unit [8]. On the transmitter side, the circuit must be capable of producing sub-nanosecond pulses. This can be achieved in different ways, such as using avalanche transistors [9,10,11], tunneling diodes [12], nonlinear transmission lines [13,14], and step recovery diodes [15,16,17]. Generators based on avalanche transistors are suitable for the generation of high amplitude pulses but not for high repetition frequencies (PRF). Furthermore, the avalanche effect of the transistor is not strict time repeatable, which leads to an uncontrolled pulse generation. A pulse generator using tunneling diodes is capable of producing a pulse duration below 100 picoseconds, but the amplitude is limited. Most of the pulse generators use step recovery diodes (SRD), which can produce a pulsewidth in the range of 100 picoseconds. Step recovery diodes are also used commonly at the receiver side in sampling heads (mixers) as strobe generators.

The second important and more complicated part of the UWB radar is the receiver. The receiver must receive and digitalize wideband signals in the order of a few GHz. One could use a high sample rate analog to digital converter (ADC) for the direct digitalization of the received signals, and such ADCs must have sample rates above ten GSa/s. Texas Instruments [18] offers an ADC up to 10 GSa/s, however, high sampling rate ADCs are too expensive for use in UWB radars and produce large amounts of data in the orders of tens and more GB per second [19]. To overcome that drawback of direct AD sampling, equivalent sample time (EST) samplers are used instead of high sample-rate ADCs [20]. This kind of sampler captures a short part of a signal in each period, and then combines them to reconstruct the original signal. EST samplers cannot capture the whole signal in one shot, and therefore need many repetitions. With this technique, the sampler stretches the original signal in the sub-nanosecond order to micro or milliseconds orders. A stretched signal is easy to digitalize with inexpensive commonly used ADCs.

The development in software defined radios (SDR) has shown that, besides the major use in communications, high bandwidth boards also open the possibility of short-range radar implementation. The SDRs can provide a quick confirmation for the proof of concept, but, in many cases, they have a disadvantage in efficiency and mobility. However, software defined radios with high bandwidth are very expensive in comparison to the proposed radar and limited in bandwidth [21,22,23].

In this paper, we propose the design of a low-cost UWB radar open platform. The novel design simplifies the picosecond pulse generator and sampler, using widely available low-cost electronic components. On the transmitter side, we use a dual SRD pulse generator, and on the receiver side, an equivalent sampling time sampling mixer, also using dual SRD as a strobe generator. The classic pulse generator is improved by removing the negative power supply, and, therefore, a pulse generator consists of a smaller number of components. The improved strobe generator used in the sampling mixer makes the sampling mixer much simpler. The used commercially available balun for splitting the strobe signal in the improved sampling mixer has a smaller signal loss in comparison to a radial slot stub balun. For the synchronization of transmitter and receiver, we use a precise fractal clock generator from Texas Instruments. The heart of a UWB radar system is the powerful open source platform Red Pitaya (RP). Transmitter, receiver, and clock are fabricated in a single PCB (printed circuit board) and could be attached directly on the RP extension headers. The advantage of our design is the modularity of the radar system. An end user could attach power amplifiers and antennas in dependence of the desired application. Received signals could be processed on any platform, due to the TCP/IP (transmission control protocol/internet protocol) connectivity of the Linux-based microcontroller called Red Pitaya.

The rest of the paper is organized as follows. Section 2.1 describes the development of a picoseconds’ generator using SR diodes. Section 2.2 describes the simulation and design of the sampling mixer and proposes the PCB outline. The next section presents the integration of the radars’ components on a single board which is attached on the RP as a daughter board. Section 4 describes the developed software and graphical interface for the radar, and the last section concludes the paper.

## 2. Design of the Proposed UWB Pulse Radar

**Figure 1:**
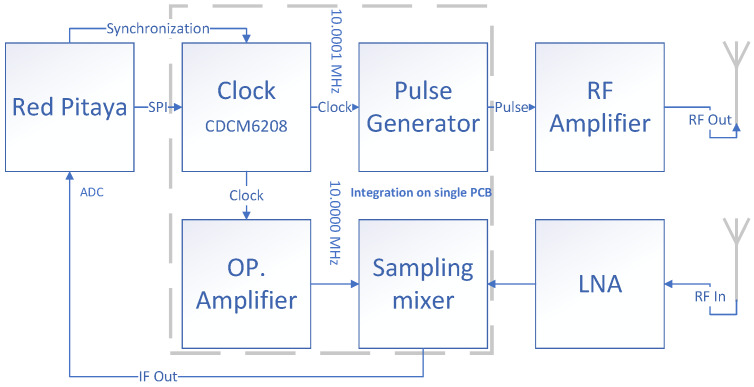
Block diagram of proposed radar. Blocks inside the dashed line are integrated on a single PCB (printed circuit board).

A block diagram of the proposed radar is depicted in Figure 1. A pulse-based radar consists of two main parts: (i) a pico-second pulse generator, and (ii) a receiving part (sampling mixer) which captures the impulse response signal. The common clock generator distribution system is used to synchronize transmitter and receiver. The sampler requires an amplified clock using an operational amplifier. A Red Pitaya is used for on board signal processing, radar manipulation, and data transmitting to host the computer using the TCP/IP protocol. A detailed explanation of each block in Figure 1 is described in the following sections.

### 2.1. Development of the Sub-Nanosecond Pulse Generator

This section presents a pulse-based generator using step recovery diodes (SRD), due to its simple implementation and ability to produce pulses in the range of hundreds of picoseconds with an amplitude of 500 mV. The authors report many different SRD generator designs. Most of them used a single SRD and pulse-shaping network for tuning the duration of the pulse. Our design is a simplification of the design proposed in [17].

**Figure 2:**
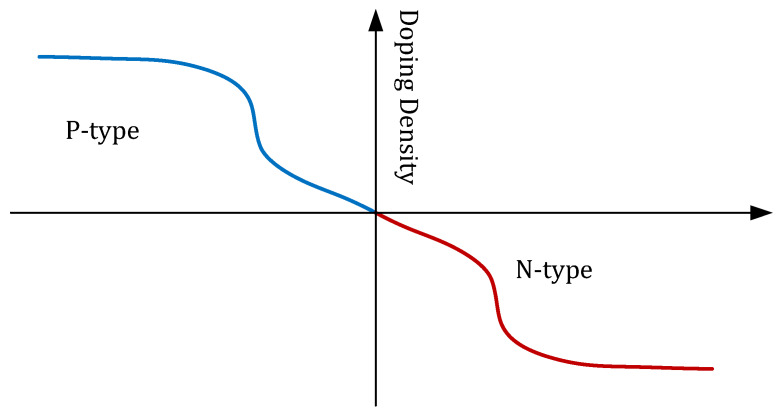
PN-junction doping profile of step recovery diode.

The SRD is a semiconductor with specific doping. The doping density is extremely small near the junction area. Thus, the density of charge carriers is also low near the junction. In other words, charge storage is negligible near the junction, which leads to fast switching from the ON to OFF state. The SRD is constructed like an ordinary PN diode. The major difference is the doping intensity along the PN junction. The doping profile is depicted in Figure 2.

The transient behavior of a PN junction is its response to a step voltage from the forward to the reverse bias regimes. Initially, the PN junction is forward biased. When the voltage of a step reverses the polarity, the junction is not switched off immediately, but takes storage time to neutralize the charges stored in the depletion region and raise the junction barrier progressively. During the storage phase, the junction holds low resistance. Near the end of the storage phase, the raising junction barrier increases the resistance, finally switching off the junction for a decay time, which is extremely short (in the picosecond range).

**Figure 3:**
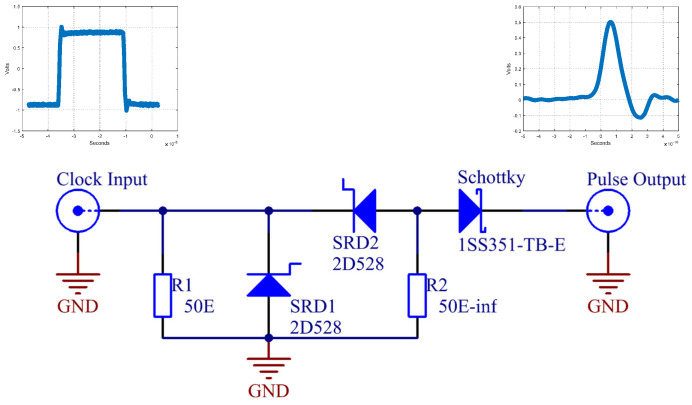
Proposed pulse generator with two SR diodes. The above schematics are measured signals at input and output, respectively.

The generator proposed in [17] consists of two SRDs, and pulse width is controlled by the change of resistance of $R_{2}$. The generator in [17] used an additional negative supply voltage. Instead of this negative supply voltage, we used a precise clock generator with symmetric ±0.5 V amplitude, and 500 ps rise time. The PIN diode on input was also removed, due to this simplification. The PIN diode in the original research was used for blocking negative input voltage. SR diodes were biased with an adjustable negative supply voltage in order to push SRDs into a reverse regime. Figure 3 shows a simple picosecond pulse generator using two SR diodes. Changing the value of resistor $R_{2}$ increases or decreases the pulse duration.

**Figure 4:**
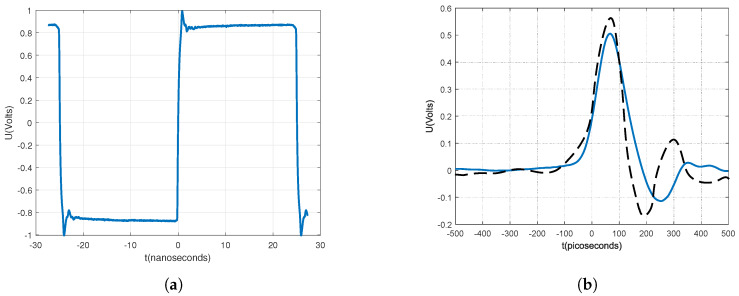
Input and output generator signals, measured with a high sampling rate oscilloscope. (**a**) Clock signal on generator input. (**b**) Generated pulse (blue line) at output in comparison with the pulse (dashed black line) from [17].

The pulse generator is driven by a precise clock generator with fractional dividers, manufactured by [24]. The clock generator is capable of producing various fractional frequencies at outputs, and so we also used it for the sampler on the receiving side, which will be discussed later. Figure 4 shows the measured clock signal at the input of the generator and measured pulse at the output of the generator. The measured pulse width is 112 ps. Figure 4b also shows the pulse from [17] for comparison. Both pulses are similar, expect our pulse has lower side ringing.

### 2.2. Development of the Sampling Mixer

The design of a sub-nanoseconds pulse generator is much simpler compared to design of the UWB receiver. The 120 picosecond pulse has as a bandwidth of about 8 GHz, which means that the receiver must be able to convert the 8 GHz bandwidth of the analog signal to digital. ADCs with tens of GSa/s are available but extremely expensive. The solution is to use an equivalent sample time ADC. A realistic approach is to sub-sample the RF signal upon extending its time scale from picoseconds into microseconds. Thus, the extended time scale of the RF signal can be handled by conventional ADCs. In the literature, the sampling mixer is also called a sampling head, or signal stretcher. In this paper, we call it a sampler. There are many different sampling concepts, and a sampler proposed in [25] is improved in this paper.

The basic principle of sampling is based on the repeated quasi-instantaneous capturing of a time-varying signal using a sampling gate. The gate is opened and closed by a time precise train of narrow strobe pulses. During sampling, repetitive scattered pulses are applied to the gate from the receiving antenna with a pulse repetition frequency (PRF) $f_{0}$. Strobe pulses on the sampling gate are triggered with the slightly offset frequency given by $f_{0}\pm \Delta f$. The received RF signal and strobe signal are mixed in a way that the strobe signal scans across the sampled RF signal. The speed of the complete sampling cycle is defined by ${}^{1}{/}_{\Delta f}$. An extending factor α is defined as ${}^{f_{0}}{/}_{\Delta f}$, and is equal to an extending ratio between the original received signal and the reconstructed signal.

**Figure 5:**
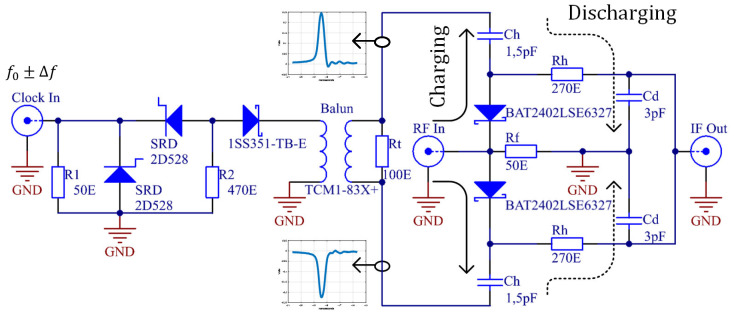
Schematic of the proposed sampling mixer. Left from the balun is the pulse generator part from the previous section. After the balun, which split the pulse from the generator into opposite pulses, is a sampling bridge with two diodes. The graphs present measured pulses at the balun’s output.

The sampler is based on a two-diode bridge. Figure 5 shows the schematic of the developed sampler. The values of components are based on simulation results using the ADS simulation tool. Furthermore, the whole PCB was designed and simulated on this simulation tool. The diodes in the bridge are low capacitance (C=0.2 pF) RF Schottky. Its low barrier height, small forward voltage and low junction capacitance make BAT24-02LS a suitable choice for mixer and detector functions in applications with frequencies which are as high as 24 GHz [26].

**Figure 6:**
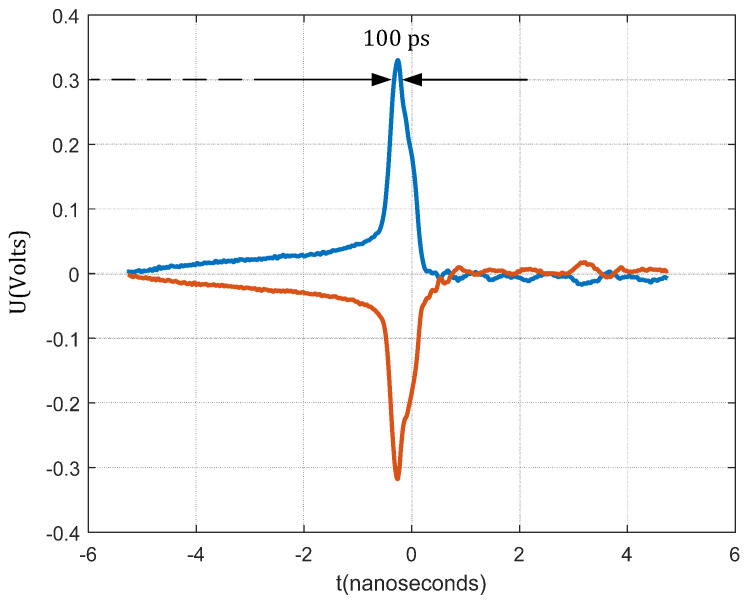
Measured strobe 100 ps pulse duration at diode forward voltage, when the diode opens the bridge, and sample input RF signal. Measurements were taken on the balun’s output.

The main challenge in the construction of a sampler is to achieve very short strobe opposite pulses with high amplitudes. The amplitude of the pulse must be able to open the bridge diode, in our case, at least 0.25 V. For generating pulses, we used the two SRD generators, described in Section 2.1. The SRD generator, triggered with an amplified clock signal [24], produced a pulse with an amplitude of approximately 1.2 V. This pulse was then divided into two pulses with opposite polarities using the balun. We chose an SMD-fabricated balun manufactured by the Mini-Circuit company (Brooklyn, NY, USA). A positive pulse opens the upper diode and a negative pulse opens the lower diode, according to Figure 5. The TCM1-83X+ balun has a wide frequency range from 10 to 8000 MHz [27]. Measured pulses on the balun output are shown in Figure 6. At around 0.3 V, where the barrier of the diode is, the pulse duration is about 100 ps (measured with an oscilloscope). In other words, the sampler bridge is capturing around 100 ps of the RF input signal in one repetition. This part of the energy is stored in capacitor $C_{d}$.

The captured RF signal is stored in the charging capacitor $C_{h}$ and, together with the series resistance of the diode ($R_{s}=8$ Ohms), provides an RF charging time around 12 ps. Resistor $R_{h}$ and capacitor $C_{d}$ provide a discharging RC network with a time constant much slower than the time constant of the charging network. Values of $C_{d}$ and $R_{h}$ were determined using a simulation tool in order to obtain good signal conversion loss.

### 2.3. PCB Design

**Figure 7:**
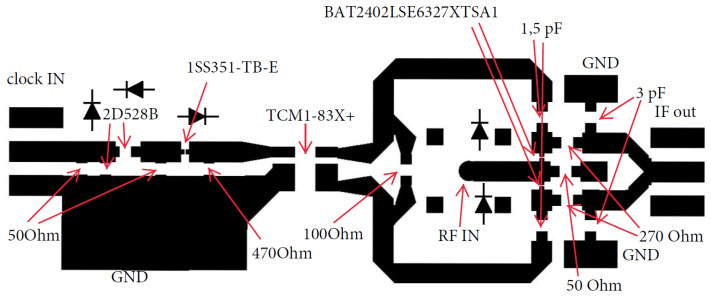
Simulated outline of sampler PCB, developed from the schematic in Figure 5.

The next step after the successful simulation of the sampler was the development of the printed circuit board (PCB). When designing an RF PCB, the designer must take into account many parameters, such as track width and length, thickness of the board, dielectric constant of the material, shape of the corners, and so forth. To produce such a PCB takes many iterations without using a simulation tool. We used the ADS simulation tool from Keysight [28] in order to produce the simulation of the whole outline of the PCB. Figure 7 shows the designed and simulated outline of the sampler.

**Figure 8:**
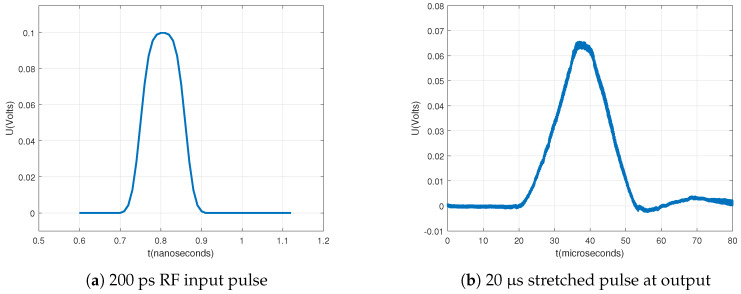
Simulation result of sampler.

In the simulation, we simulated a 200 ps width pulse at the input RF port (denoted as RF IN in Figure 7). The input pulse had a peak of voltage 0.1 V and a rise/fall time of 0.1 ns. Strobe frequency $f_{0}$ was set to 10 MHz and frequency Δf to 100 Hz. The sampler stretched the input pulse from 200 ps to 20 μs, which confirmed extending factor α, defined as f0Δf=10MHz100Hz= 100,000, shown in Figure 8.

**Figure 9:**
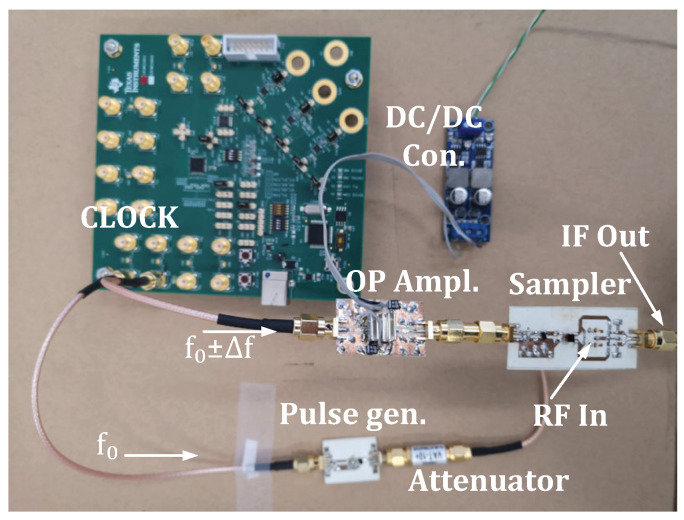
Prototype of radar without antennas ready for test measurements. Instead of an antenna, the attenuator is connected.

**Figure 10:**
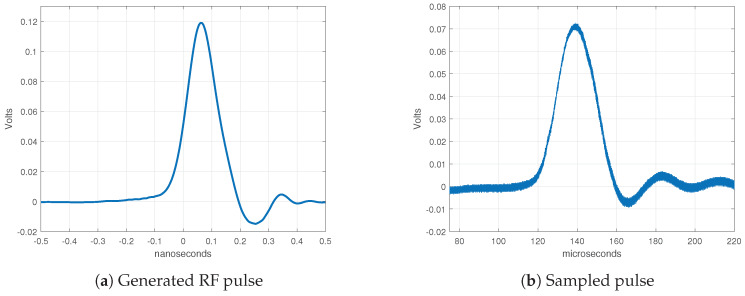
Measured results of sampler.

The PCB was fabricated using two-sided ROGERS RO4350B laminate with dielectric constant 3.66 and substrate thickness 0.762 mm. The fabricated sampler, pulse generator, and clock source are depicted in Figure 9. After fabrication, we took measurements of the sampler. A synchronized two output channels clock CDC6208 was used for generating pulses and for generating the strobe signal for sampling. Measurements are shown in Figure 10. Generated RF pulses were measured using an Agilent 40 GSa/s oscilloscope, meanwhile the IF (Intermediate Frequency) output of the sampler was measured using a Rigol 2 GSa/s oscilloscope. We can observe in Figure 10 that the sampled pulse was stretched 100,000 times, and that measurements confirmed the simulation results. The IF output of the sampler can now be digitalized using an ADC with a 5 MSa/s or less sampling rate.

**Figure 11:**
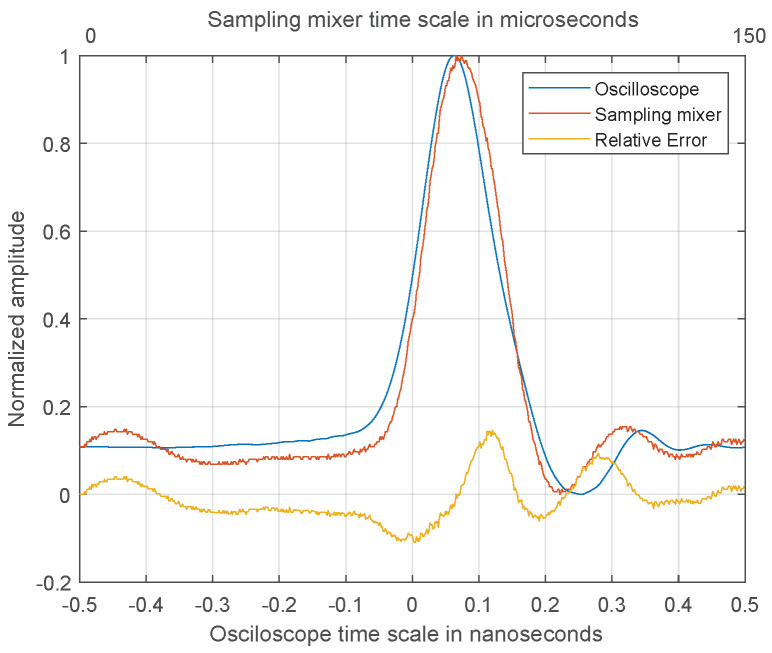
Comparison of signal acquisition between oscilloscope and sampler. The yellow curve is the relative error.

A comparison between the oscilloscope signal and sampler signal was made to validate the sampler signal acquisition. In Figure 11 are both signals (from oscilloscope and sampler), normalized and scaled. From the signals, the relative error was calculated using the equation: $e=\frac{signal_{sampler}-signal_{ocilloscope}}{signal_{ocilloscope}}$. The yellow curve in Figure 11 represents relative error. The peak value of the relative error is below 15% and the average relative error is below 1%.

### 2.4. Digitalization of the Sampler’s Output

The IF output of the sampler produced a down converted RF signal. We could perform signal capturing using a classic oscilloscope, but it is not practical for radar applications. To draw a B-scan or image from radar signals, it is necessary to use signal processing. Low cost microcontrollers are not powerful enough to capture and process signals. Furthermore, their ADCs have a sample rate up to 1 MSa/s, which is not enough. After research of the market we chose a signal processing platform named Red Pitaya, which is low cost and has a high performance. On the analog sidem, RPs have two channel AD and DA converters with sample rates up to 125 MSa/s. The heart of the platform is a powerful FPGA Zynq 7010. The FPGA platform is often used for custom high speed signal processing (e.g., fast real-time signal processing, spectrometry, and video processing). The post processing can be done on-chip utilizing dual-core ARM Cortex-A9 processors. Data can be stored locally on an SD card or can be accessed remotely through ethernet or WiFi [29].

### 2.5. Synchronization of Clock and AD Conversion

RP offers triggering of an AD channel with the voltage level (the same as an oscilloscope). When we are capturing periodic signals this trigger method is appropriate. The start of acquisition is caused when the voltage level of the generated pulse exceeds the trigger voltage level. Since the pulse shape is slightly changing during the whole acquisition time, this causes the captured data to not be aligned, and it is impossible to draw a useful B-scan. Another option is to trigger a time delay, but there is an issue in the phase of generating two clocks. When the clock starts, the outputs of two frequencies begin with the same phase, and, therefore, we can first capture the period of signal using a time delay trigger. The second period is not in the phase anymore, and, therefore, the synchronization is lost. Luckily, the CDCM6028 has the option of a re-synchronization of the clocks. That gave us the idea to synchronize the clock and trigger the ADC channel, since RP offers analog outputs which are capable of generating phase synchronized pulse width modulations (PWM). We used one PWM output for the re-synchronization of the clock, and the other for triggering the AD conversion. Tests showed us that this solution works well.

## 3. Integration of Radar’s Components

The first prototype of the radar was modularity development. Each part was on a separate PCB, due to better measurements and testing. When the tests of radar components were done, the integration of components was started in order to minimize the radar. We integrated the clock CDCM6208, SRD pulse generator, sampler, and some power supply on a single PCB. The PCB was designed to attach to RP connectors as a daughter board. We did not place the output RF amplifier and input LNA (low noise amplifier) on the PCB due to the latter’s flexibility. On the integrated PCB are also digitally controlled power supplies for supplying various RF amplifiers and LNAs.

**Figure 12:**
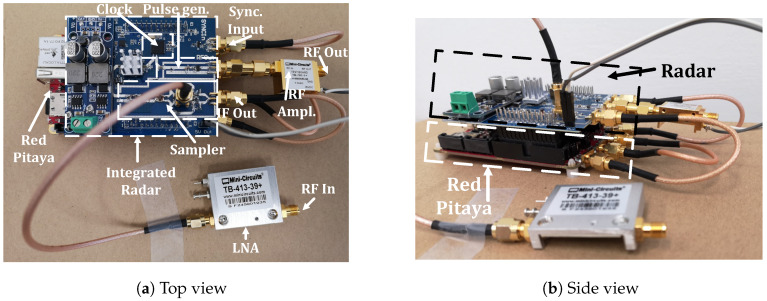
Picture of the proposed radar attached on the Red Pitaya micro-controller.

The sampler and pulse generator are placed on the PCB and RF and isolated using a via fence. Other RF design constraints were also considered, such as 50 Ohm lines, good ground connections, and so forth. The radar was fabricated on the same laminate as the prototype. Figure 12 shows the integrated radar attached on the RP board.

## 4. Development of Radar GUI

In the previous section we described the hardware for the proposed radar. We also developed complete software, including a Graphical User Interface (GUI). Since the RP does not include a display or a display port, we used a laptop or desktop computer for the representation of the data obtained from the radar. There are many options to take data from RP, such as ethernet, WiFi, USB, or via SD card. The best way is to use ethernet, since a Linux operational system is installed on the RP. The software for RP is completely open source and offers API (Application Programming Interface) functions to control analog inputs and outputs. Programs for RP can be made in ansi C, Python, Matlab, and Labview programming languages.

**Figure 13:**
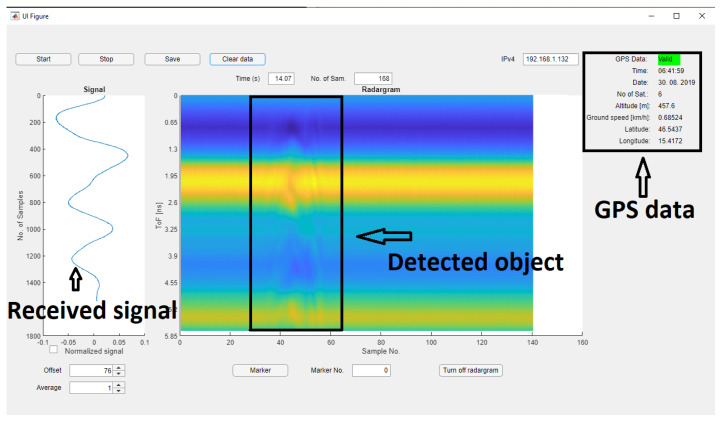
GUI of the radar. On the left side is a signal captured by the sampler. On the right side are captured signals joined into a figure (B-scan).

The software for our radar is divided into two parts: (i) software which runs on the RP and (ii) software on the laptop. The RP software was developed in ansi C and controls the clock module via SPI (Serial Peripheral Interface), captures analog data from the AD channel, generates PWM for clock synchronization, and communicates over TCP/IP with the host computer/laptop. Last but not least, the software also supports a GPS module controlled over the NMEA (National Marine Electronics Association) protocol. The RP software captures raw data on the ADC channel and sends it over TCP/IP to the host computer. On the host PC is a developed GUI with signal processing for radar data presentation. We used MATLAB App Designer for programming the GUI [30]. The graphical interface of the radar is depicted in Figure 13. A user can set the IP address of the radar, start and stop signal capturing, put a visible marker on a B-scan, save all capturing data for further processing, and set the vertical offset of the signal, and so forth.

Figure 13 presents a radargram. On the left side in Figure 13 is the signal from the receiver antenna obtained by the sampler and stretched 100,000 times. Originally, the amplitude (x-axis) is in voltage, but the user could switch to a normalized amplitude. On the y-axis are a number of samples obtained with the Red Pitaya ADC. In the current set-up, the radar captured around 10 whole signals per second. On the right side of the radar GUI is depicted an image generated from the left side signals (B-scan). Each triggered signal is real-time added on the ride side of image. The signals are colored depending on the amplitude of the signal. On the y-axis is the time of flight (ToF) calculated from the following parameters: (i) number of captured samples, (ii) sampling rate of the ADC, (iii) time stretch ratio of the sampler. From the ToF it is possible to calculate the penetrating depth of the RF signal according to the surrounding material, since the propagation velocity of an RF signal is dependent on the dielectric constant of the material.

## 5. Conclusions

This paper presents the development of a low-cost UWB pulse radar with a bandwidth from DC up to 8 GHz. The pulse generator and sampler designs were improved and implemented using commercially available low cost electronic components. An existing design of a pulse-based radar using SRD was improved by removing the negative power supply, resulting in a reduced number of electronic components. To receive ultra wide band pulses, a signal stretcher was improved by using the improved pulse generator as a strobe pulse generator, which made the design much simpler, and enabled us to acquire a received signal using a much lower sample rate. A micro controller with a Linux operating system called Red Pitaya was used for all signal manipulations, acquisition, signal processing, and communication with peripheral devices. The data transfer was made using a TCP/IP communication, and a simple GUI was developed for data visualization.
